# SPIKES: Identification of physicochemical properties of spike proteins across diverse host species of SARS-CoV-2

**DOI:** 10.1016/j.xpro.2022.101460

**Published:** 2022-05-24

**Authors:** Srinivasulu Yerukala Sathipati, Ming-Ju Tsai, Tonia Carter, Sanjay K. Shukla, Shinn-Ying Ho

**Affiliations:** 1 Center for Precision Medicine Research, Marshfield Clinic Research Institute, Marshfield, WI, USA; 2 Hinda and Arthur Marcus Institute for Aging Research at Hebrew Senior Life, Boston, MA, USA; 3 Department of Medicine, Beth Israel Deaconess Medical Center and Harvard Medical School, Boston, MA, USA; 4 Institute of Bioinformatics and Systems Biology, National Yang Ming Chiao Tung University, Hsinchu, Taiwan; 5 Department of Biological Science and Technology, National Yang Ming Chiao Tung University, Hsinchu, Taiwan; 6 Center for intelligent Drug Systems and Smart Bio-Devices (IDS^2^B), National Yang Ming Chiao Tung University, Hsinchu, Taiwan; 7 College of Health Sciences, Kaohsiung Medical University, Kaohsiung, Taiwan

**Keywords:** Bioinformatics, Microbiology, Proteomics, Systems biology

## Abstract

We describe a protocol to identify physicochemical properties using amino acid sequences of spike (S) proteins of SARS-CoV-2. We present an S protein prediction technique named SPIKES, incorporating an inheritable bi-objective combinatorial genetic algorithm to determine the host species specificity. This protocol addresses the S protein amino acid sequence data collection, preprocessing, methodology, and analysis.

For complete details on the use and execution of this protocol, please refer to Yerukala Sathipati et al. (2022).

## Highlights

• Identify informative physicochemical properties of the SARS-CoV-2 spike protein
• SPIKES predicts the species specificity across diverse host species of SARS-CoV-2
• This protocol addresses spike protein sequence retrieval and analysis procedures
• Differences exist in the S protein properties of diverse host species CoVs

## Abstract (editor highlights)

Publisher’s note: Undertaking any experimental protocol requires adherence to local institutional guidelines for laboratory safety and ethics.

## Graphical abstract

**Figure:**
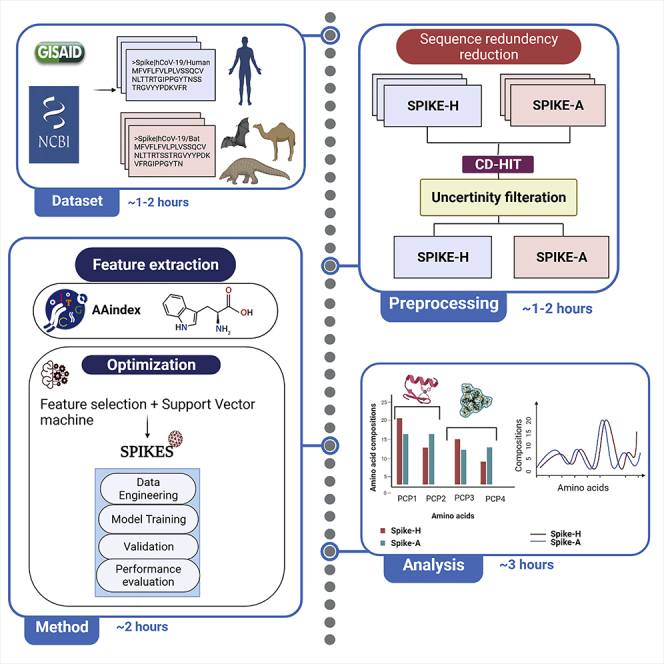

## Before you begin

We describe the protocol to identify physicochemical properties (PCPs) of Spike (S) proteins of severe acute respiratory syndrome coronavirus 2 (SARS-CoV-2) using the SPIKES computing platform. Here, we used amino acid sequences of S proteins of human and animal host coronaviruses (CoVs) to determine the species specificity. This protocol demonstrates the workflow of the SPIKES to identify informative PCPs of S proteins using amino acid sequence-based properties retrieved from the AAindex by Kawashima et al. (Kawashima et al., 2008). There are three major parts in this protocol, including, 1) dataset construction and preprocessing, 2) feature extraction and modeling, and 3) PCP analysis. The detailed steps involved in the protocol are shown in the Graphical abstract. The following steps explain the use of the SPIKES program and parameters to carry out the experiment.

1. The software specifications to use the SPIKES program are provided in the key resources table.
2. The R software environment and Perl program are required for this protocol. The latest R version (4.1.2) can be downloaded from link and R Studio from link. Perl can be downloaded from link.
3. LIBSVM, a library of support vector machine (SVM), is necessary to run the SPIKES model files. The v3.20 version of the LIBSVM library in the C++ environment can be downloaded from link.
4. The SPIKES prediction pipeline can be downloaded from GitHub: https://github.com/mingjutsai/SPIKES

**CRITICAL:** To download the initial dataset, computer must have ∼4 GB of disk space. The minimum requirements to run the protocol are 8 GB RAM and 1.5 GHz quad-core processor. The SPIKES program can be performed in Mac OS/Linux platforms.

### Overview of the method

#### Dataset preparation

**Timing: ∼1–2 h**

5. The S protein sequences of the human and animal host’s CoVs can be downloaded from the Global Initiative on Sharing Avian Influenza Data (GISAID) link and National Center for Biotechnology Information (NCBI) link databases.
  a. Human and animal host coronavirus (CoV) protein sequences in FASTA format can be downloaded from GISAID and NCBI databases. Examples of sequence IDs of human and animal host CoVs and their accession links are shown in Table 1. The dataset consisting of human and animal host CoV sequences are available at Github link. The description of the FASTA format can be found at link.

**Table 1:** The human and animal host spike protein sequences and their available sources

Spike protein sequence	Example sequence ID	Availability
Human host	Spike|hCoV-19/Wuhan/WIV04/2019|2019-12-30|EPI_ISL_402124|Original|hCoV-19ˆˆHubei|Human|Wuhan	link
Animal host	AAY88866.1 spike glycoprotein [Bat SARS coronavirus HKU3-1	link
  b. Reduce the sequence redundancy and ambiguity of the sequences using CD-HIT web server (Huang et al., 2010) link.

**CRITICAL:** The time needed depends on the data size, computer processing time and disk storage. Please check the recommended computer specifications before downloading and extracting the data from the GISAID website.

#### Feature extraction and modeling

**Timing: ∼ 1 h**

6. Convert the FASTA sequences to a numerical dataset.
  a. Extract the PCPs from the FASTA sequences using the Amino acid index database (AAindex).
  b. Extract amino acid compositions, dipeptide compositions, and pseudo amino acid compositions from the S protein sequences for the purpose of analysis.
7. Scaling the features is suggested before you start the data modeling.
8. Divide the dataset into training and test sets for model training and evaluation, respectively.
9. Use SPIKES for feature selection and model tuning.
  a. SPIKES is designed to learn the best combination of feature set and model parameters from the applied data set. SPIKES selects a subset of features from the original data and predicts the species specificity simultaneously.
  b. There are two parts in this section, first, the setting of parameters for feature selection and evaluation, and second, the setting of the search range of SVM parameters.
    i. The GA-chromosome consists of binary GA-genes for selecting informative features of PCPs and two 4-bit GA-genes for encoding the parameters *C* and *γ* of SVM. The inheritable bi-objective combinatorial genetic algorithm (IBCGA) can simultaneously obtain a set of solutions, *Xr*, where *r*=*r*_*end*_, *r*_*end*_ + 1,…, *r*_*start*_ in a single run. In SPIKES, the radial basis function (RBF) kernel was used for the implementation of SVM.
    ii. The prediction model with tuned parameters is available at link.
  c. SPIKES identifies informative features that can distinguish the S proteins of humans and animal CoVs.

**CRITICAL:** Machine learning knowledge and programming skills are necessary to tune the parameters of SVM.

**Trouble shooting**: A stepwise procedure and a set of recommended parameters for the SPIKES model can be accessed from link.

#### Physicochemical property analysis

**Timing: ∼ 3 h**

10. Use Microsoft Excel or GraphPad Prism 9 to calculate the amino acid differences between human and animal host coronaviruses.
  a. Measure the amino acid compositions for all S protein sequences of human and animal host CoVs using the code provided in the following pages.
  b. Compare the amino acid composition differences between S proteins of human and animal host CoVs using the subtraction function in Excel (example [Human(A)– Animal(A)]).
  c. The expected outcome would be the differences between amino acid compositions between S proteins of human and animal host CoVs.
11. Measure the distinguishing features, identified by SPIKES, in the S proteins of human and animal CoVs to determine how amino acid preferences differ between the S proteins of human and animal CoVs.
12. Use the spike glycoprotein mutation surveillance dashboard from CoVsurver in GISAID to explore mutations in the CoV variant of interest.
13. Download and install UCSF Chimera software for protein structure visualization from link.

## Key resources table

**Table:** 

REAGENT or RESOURCE	SOURCE	IDENTIFIER
**Deposited data**		

Spike protein sequences	Global Initiative on Sharing Avian Influenza Data	GISAID - Initiative
Spike protein sequences	National Center for Biotechnology Information	National Center for Biotechnology Information (nih.gov)

**Software and algorithms**		

Support vector machine	Chang and Lin (2011)	LIBSVM -- A Library for Support Vector Machines (ntu.edu.tw)
Sequence redundancy	Huang et al., 2010	http://weizhong-lab.ucsd.edu/cdhit-web-server/cgi-bin/index.cgi
Perl	Perl program	https://www.perl.org/get.html
R studio	The R project	https://www.rstudio.com/products/rstudio
Protein structure visualization	UCSF Chimera	https://www.rbvi.ucsf.edu/chimera/
Inheritable bi-objective combinatorial genetic algorithm	Ho et al. (2004)	Inheritable genetic algorithm for biobjective 0/1 combinatorial optimization problems and its applications - PubMed (nih.gov)
SPIKES methods	This paper	https://github.com/mingjutsai/SPIKES https://doi.org/10.5281/zenodo.6502505
Supplemental information	This paper	https://data.mendeley.com/datasets/6s9wt7zzxz/1 https://doi.org/10.17632/6s9wt7zzxz.1

## Step-by-step method details

### Data collection and preprocess

**Timing: ∼2 h**

This section describes download of the S protein sequence data from GISAID and NCBI databases.

1. User registration is required to download the data from GISAID link.
  a. First, log in to the GISAID database, search for the SARS-CoV virus in the search box. Select the host (human/others), and download the S protein sequences in FASTA format.
  b. Human and animal host CoV S protein sequences can be obtained from the NCBI database using the search terms “S protein” and “SARS-CoV”.
  c. A description of acquired data from the GISAID and NCBI databases, and an example of the FASTA file format is shown in Figures 1A and 1B.

**Figure 1:**
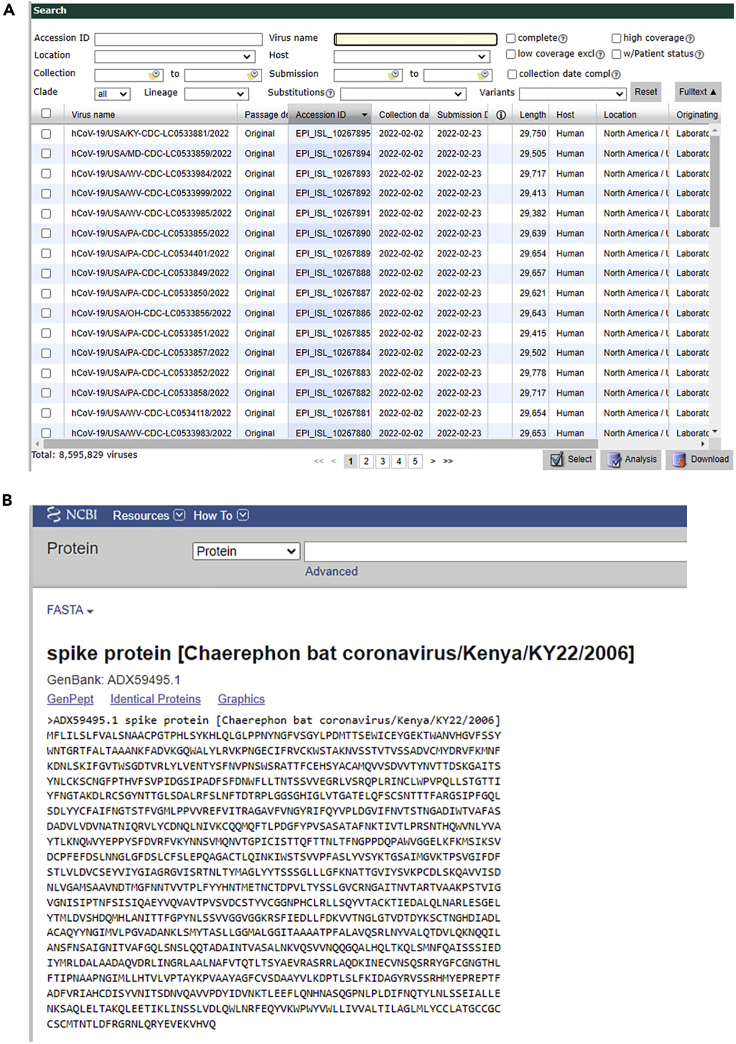
Screenshots showing spike protein data acquisition from databases (A and B) (A) Displaying SARS-CoV-2 data from GISAID and (B) NCBI databases. An example of amino acid sequence in FASTA format.
2. Preprocessing the FASTA sequence data.
  a. The human and animal FASTA sequences must be formatted independently.
  b. The input must be all the S protein sequences of human/animal in bulk.
  c. The Initial dataset consisted of several thousand S protein sequences of spike human (spike-H) and spike animals (spike-A). Because amino acid changes are a crucial factor for disease transmission, we considered 99% sequence identity in the redundancy reduction process. Use the CD-HIT web server to reduce the sequence redundancy.
    i. Go to the CD-HIT we bserver at link.
    ii. Load query FASTA sequence file under cd-hit tab.
    iii. Choose sequence identity cut-off value of 0.99.
    iv. Algorithm parameters: select ‘yes’ to the ‘use global sequence identity’ and ‘sequence is clustered to the best cluster that meet the threshold’. Set bandwidth of alignment to 20 and length of sequence to skip to 10.
    v. Alignment coverage parameters: set a default value ‘0’ for minimal alignment coverage (fraction) for the longer sequence, minimal alignment coverage (fraction) for the shorter sequence, and minimal length similarity (fraction). Set ‘unlimited’ to the maximum unaligned part (amino acids/bases) for the longer sequence, maximum unaligned part (amino acids/bases) for the shorter sequence, and maximum length difference in amino acids/bases(-S).
    vi. User may change or use default options for algorithm parameters and alignment coverage parameters.
    vii. Click on submit button.
  d. Users can also use the protein-to-protein BLAST option from the BLAST server link to determine the sequence similarity between two protein sequences.
  e. Exclude ambiguous FASTA sequences by removing any protein sequences containing special characters, such as ‘B, J, O, U, X and Z’, that do not represent a specific amino acid.
  f. After sequence redundancy reduction and accounting for uncertainties, the dataset consisted of 211 and 611 S protein sequences of spike-H and spike-A, respectively. For analysis, select the 211 spike-H proteins and a random sample of the 611 spike-A proteins. This will produce a final balanced dataset consisting of 211 and 211 S protein sequences of spike-H and spike-A, respectively.
  g. It could take ∼2 h for downloading the desired/updated SARS-CoV-2 sequences and preprocessing, which depend on the speed of the Internet connection and the computer processor.

**CRITICAL:** Ensure you have sufficient storage on your computer before downloading data from GISAID. Downloading and extracting the initial dataset from GISAID requires ∼ 4 GB of disk space.

### Feature extraction

**Timing: ∼1 h**

The AAindex database contains numerical indices representing the PCPs of amino acids (Kawashima et al., 2008). Each index represents a different PCP and has 20 values, one for each of the 20 naturally occurring amino acids.

3. SPIKES adopted 531 PCPs retrieved from the AAindex database as candidate features to distinguish S proteins of diverse CoV strains. The original CoVs’ amino acid sequences were converted into AAindex numerical indices according to the 531 PCP values. That is, each amino acid in the sequence was represented by the 531 index values for that amino acid in the AAindex. The feature representation of the 531 PCPs is described as follows:
  a. Collect the spike protein sequences from the dataset.
  b. Calculate the amino acid composition *l*(*aa*_*i*_) of a sequence for the *i*^th^ amino acid *aa*_*i*_ of 20 amino acids and encode the protein sequence of variable length into the feature vector with a length of 531 properties. The AAindex matrix is provided in link.
  c. Calculate the feature value of the *p*^*th*^ physicochemical property, PCP(*p*), of a spike protein, where *p*=1, 2, …, 531.

(Equation 1) $$PCP\left(p\right)=\sum_{i=1}^{20}l\left(aa_{i}\right).PCP_{p}\left(aa_{i}\right)$$

4. Before converting the FASTA sequence into AAindex features, users are required to compile the AAindex to get the executable program.

>git clone https://github.com/mingjutsai/SPIKES

>cd SPIKES

>src/aaindex

>make

### Feature selection and model tuning

**Timing: ∼1 h**

Feature selection and model tuning are the core of this workflow.

5. Feature scaling helps to normalize the features within a specific range. Scaling maps the feature values into the {-1, 1} or {0, 1} interval. The feature scaling to the dataset can be applied using “svm-scale”, a regular program provided by LIBSVM.
6. The feature values were scaled in to the range of -1 and +1. The feature scaling of the dataset is provided in the link.

>cd SPIKES

>src/libsvm_320

>make

Usage: svm-scale [options] data filename

 Options

 -l lower : x scaling lower limit (default -1)

 -u upper : x scaling upper limit (default +1)

 -y y_lower y_upper : y scaling limits (default: no y scaling)

 -s save_filename : save scaling parameters to save_filename

 -r restore_filename : restore scaling parameters from restore_filename

***Note:*** The training and test data should apply the same scaling parameter. For more information, please refer LIBSVM website and download its SVM package: link.

### Feature selection

**Timing: ∼2–3 h**

7. The SPIKES was developed using SVM (Vapnik, 1999) incorporating an optimal feature selection algorithm, IBCGA (Ho et al., 2004) to select informative features from a large number of candidate features. SPIKES selects important PCPs of S proteins and distinguishes the human host from the animal host coronaviruses simultaneously. Further details of the feature selection algorithm could be found in these studies (Tung and Ho, 2007) and (Tung and Ho, 2008). The parameter tuning of SVM can also be found in the study (Yerukala Sathipati and Ho, 2021). The steps involved in the feature selection process is depicted in Figure 2.

**Figure 2:**
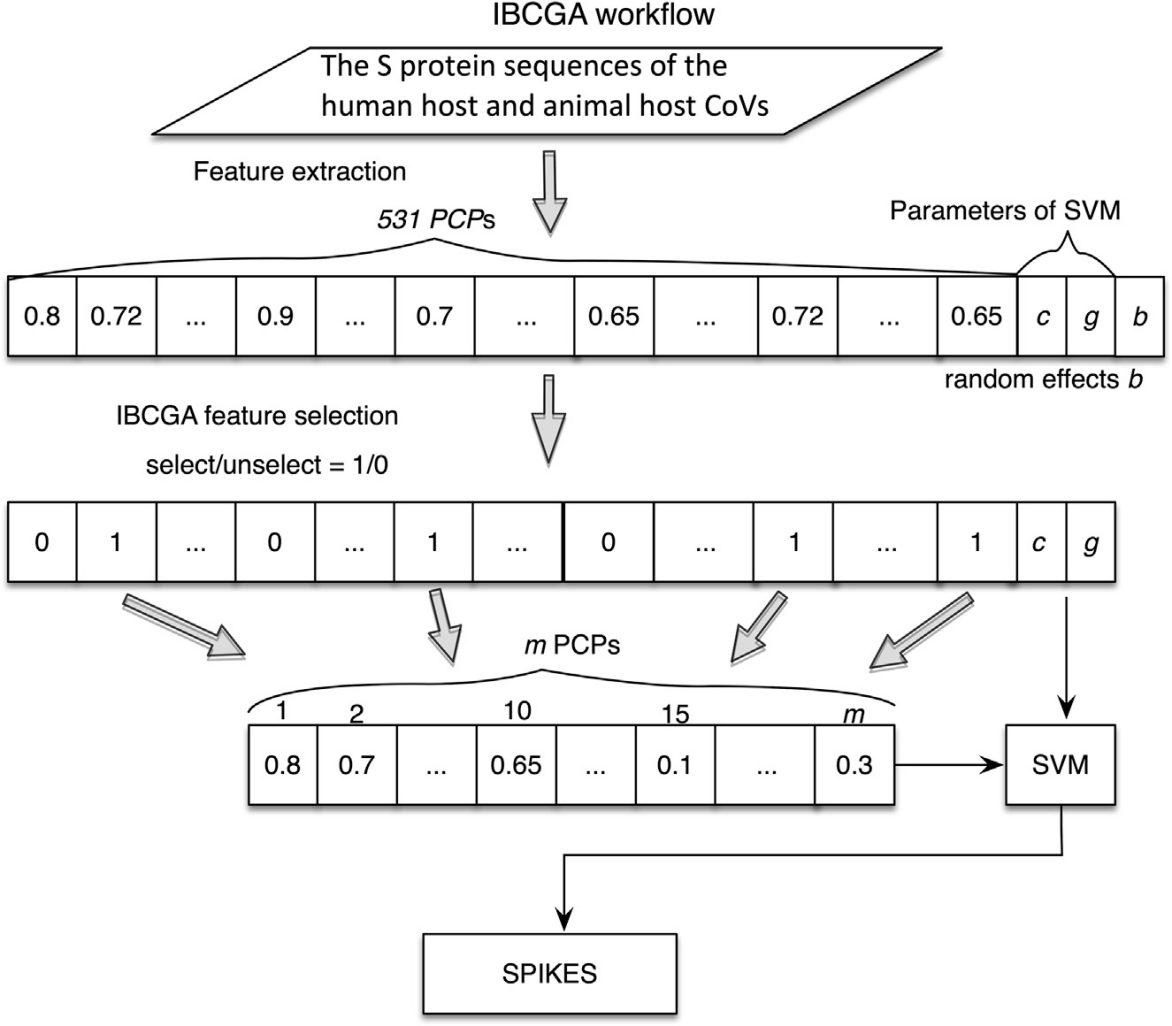
The steps involved in feature selection algorithm
8. The steps involved in IBCGA are as follows:
  a. ***Step 1***: (Data Preparation) Compile the training sets from spike-H and spike-A for developing and evaluating the SPIKES method.
  b. ***Step 2***: (Initialization) Randomly generate an initial population of *N*_*pop*_ individuals. *N*_*pop*_ = 50, *r*_*start*_ = 50, *r*_*end*_ = 10, and *r* = *r*_*start*_.
  c. ***Step 3***: (Evaluation) Evaluate the fitness value of all individuals using the fitness function that is the prediction accuracy in terms of 10-fold cross-validation (10-CV).
  d. ***Step 4***: (Selection) Use a conventional tournament selection method that selects the winner from two randomly selected individuals to generate a mating pool.
  e. ***Step 5***: (Crossover) Select two parents from the mating pool to perform an orthogonal array crossover operation.
  f. ***Step 6***: (Mutation) Apply a conventional bit mutation operator to GA-genes of SVM parameters and a swap mutation to the binary GA-genes for keeping *r* selected features. The best individual was not mutated for the elite strategy.
  g. ***Step 7***: (Termination test) If the stopping condition for obtaining the solution *X*_*r*_ is satisfied, output the best individual as the solution *X*_*r*_. Otherwise, go to step 2.
  h. ***Step 8***: (Inheritance) If *r* > *r*_*end*_, randomly change one bit from 1 to 10 in the binary genes for each individual. Decrease the number r by one and go to step 2. Otherwise, stop the algorithm.
  i. ***Step 9***: (Output) Obtain a set of *m* features in total for PCPs, from the best solution *X*_*m*_ among the solutions *X*_*r*_, where *r* = *r*_*end*_, *r*_*end*_ + 1,… , *r*_*start*_*.*
9. The SPIKES model file to select the PCPs has been provided at link.
10. First train the SPIKES-Training data using the SPIKES model file and validate on SPIKE-Test data. The usage of datasets and SPIKES is provided at link.

### Physicochemical properties identification

**Timing: ∼10 min**

We’ve built an easy-to-use prediction pipeline from protein FASTA to prediction results using 11 informative features (Table 2) prediction model.

11. Create an output folder for prediction results and use the following command to conduct the SPIKES prediction pipeline:

>mkdir output

>perl SPIKES_main.pl protein.fa output

**Table 2:** Informative physicochemical properties obtained using SPIKES

	Aaindex ID	Feature description
1	RACS820104	Average relative fractional occurrence in EL(i) (Rackovsky and Scheraga, 1982)
2	ROBB760101	Information measure for alpha-helix (Robson and Suzuki, 1976)
3	RACS820109	Average relative fractional occurrence in AL(i-1) (Rackovsky and Scheraga, 1982)
4	GEIM800105	Beta-strand indices (Geisow and Roberts, 1980)
5	QIAN880137	Weights for coil at the window position of 4 (Qian and Sejnowski, 1988)
6	PRAM820103	Correlation coefficient in regression analysis (Prabhakaran and Ponnuswamy, 1982)
7	JOND920102	Relative mutability (Jones et al., 1992)
8	NAKH920103	Amino acid composition of EXT of single-spanning proteins (Nakashima and Nishikawa, 1992)
9	OOBM850101	Optimized beta-structure-coil equilibrium constant (Oobatake et al., 1985)
10	CHAM830104	The number of atoms in the side chain labeled 2+1 (Charton and Charton, 1983)
11	ROBB760103	Information measure for middle helix (Robson and Suzuki, 1976)

### Physicochemical property analysis

**Timing: ∼2–3 h**

Use the PCPs selected by the SPIKES model for comparing features between the S proteins of human and animal CoVs.

12. Measure the PCPs for Spike-H and Spike-A to compare the property differences in human and animal host CoVs.
13. Measure the amino acid and dipeptide compositions.
  a. The values of *f*(*aa*_*i*_) were calculated for the Spike-H and Spike-A where *i*=1,…, 20. The feature set of DPC is represented as a feature vector of length 400 for the dipeptides, (i.e., AA, AC…. YY). The feature set of PseAAC is represented as a feature vector of length 80 for the AAC and PseAAC for hydrophilicity and hydrophobicity.
  b. The amino acid and dipeptide conversion can be done using the following sequence conversation code via R studio.

>library(seqinr)

> Human <- read.fasta('Human.fasta', seqtype="AA", as.string = TRUE)

>NonHuman <- read.fasta('nHuman.fasta', seqtype="AA", as.string = TRUE)

#Check for non-amino acids in the FASTA sequence

>Human[(sapply(Human, protcheck))]

>NonHuman [(sapply(NonHuman, protcheck))]

#AAC and DPC Feature extraction

AAC <- t(sapply(Human, extractAAC))

DPC <- t(sapply(Human, extractDC))

# Do the same for NonHuman host coronavirus sequences

Below is the list of PCPs obtained from SPIKES.

***Note:*** Users may get a different set of features by using a larger S protein dataset and modifying the tuning of search parameters.

14. To determine the significance of compositional changes in amino acids in S protein from different strains of CoVs, the user may use the hCoV-19 spike glycoprotein mutation surveillance dashboard from GISAID.
  a. Login into the GISAID webpage. Select EpiCoV > spike glycoprotein mutation surveillance.
15. The specific amino acid changes in S proteins have been implicated in increasing the infectivity and virulence of new variants. User may use GISAID data statistics to examine the amino acid changes in S protein that increased the infectivity in emerging new variants.
16. Users can use Chimera protein visualization software to visualize the S protein structure in .pdb format and observe the amino acid preferences in alpha helices and beta sheets.
  a. Visit the Chimera URL link and download and install the software.
  b. Visit the Protein Data Bank URL link. Search for the S protein (e.g., PDB: 6VXX).
  c. Download the protein structure in .pdb format.
  d. Open the 6VXX.pdb file in the Chimera protein visualization software. File > open > 6VXX.pdb.

## Expected outcomes

The expected outcome from SPIKES includes the prediction probability of distinguishing human and animal host CoVs. The normalized probabilities less than 0.5 are predicted to be human host spike proteins, those greater than 0.5 are animal host spike proteins. The results format is as shown on the Github page link.

**Figure 3:**
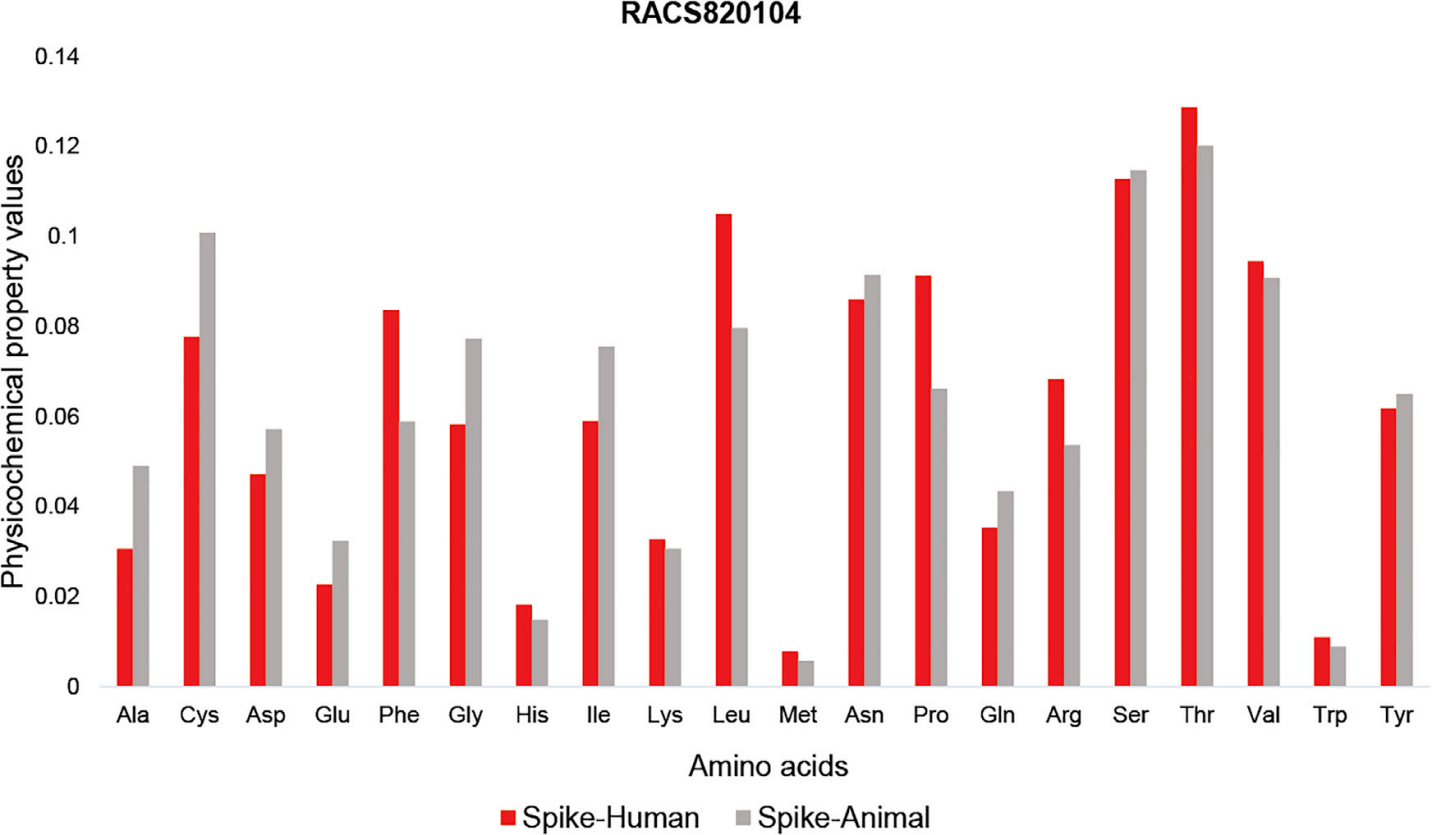
The comparison of physicochemical property (AAindex ID: RACS820104) between spike proteins of human and animal host coronaviruses The ID RACS820104 represents the average relative fractional occurrence in EL(i).

The comparison analysis of PCPs reveals the amino acid preferences for each property between human and animal host CoVs. A PCP is compared between human and animal host CoVs as shown in Figure 3. The PCP values can be calculated for the human and animal host CoVs using Microsoft Excel or Graphpad Prism.

**Figure 4:**
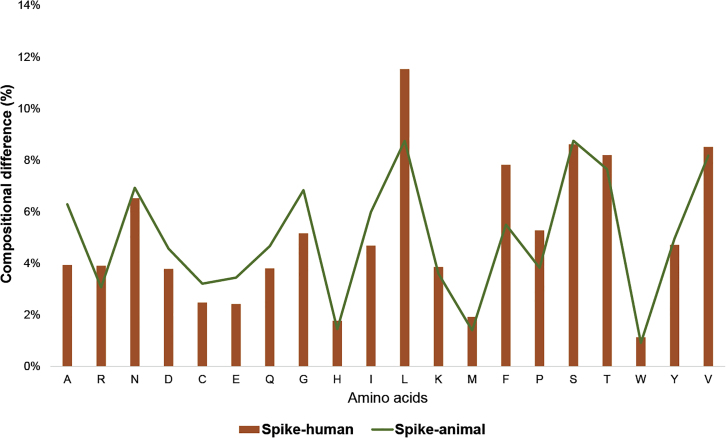
The comparison of amino acid compositions between spike proteins of human and animal host coronaviruses

PCPs could be calculated using the following code or either using an Excel sheet. In Excel enter the PCP values from the AAindex link in one column and multiply with the amino acid composition values of either S protein sequences of human or animal. Next, the amino acid composition of S proteins between human and animal host CoVs can be compared as shown in Figure 4.

**Figure 5:**
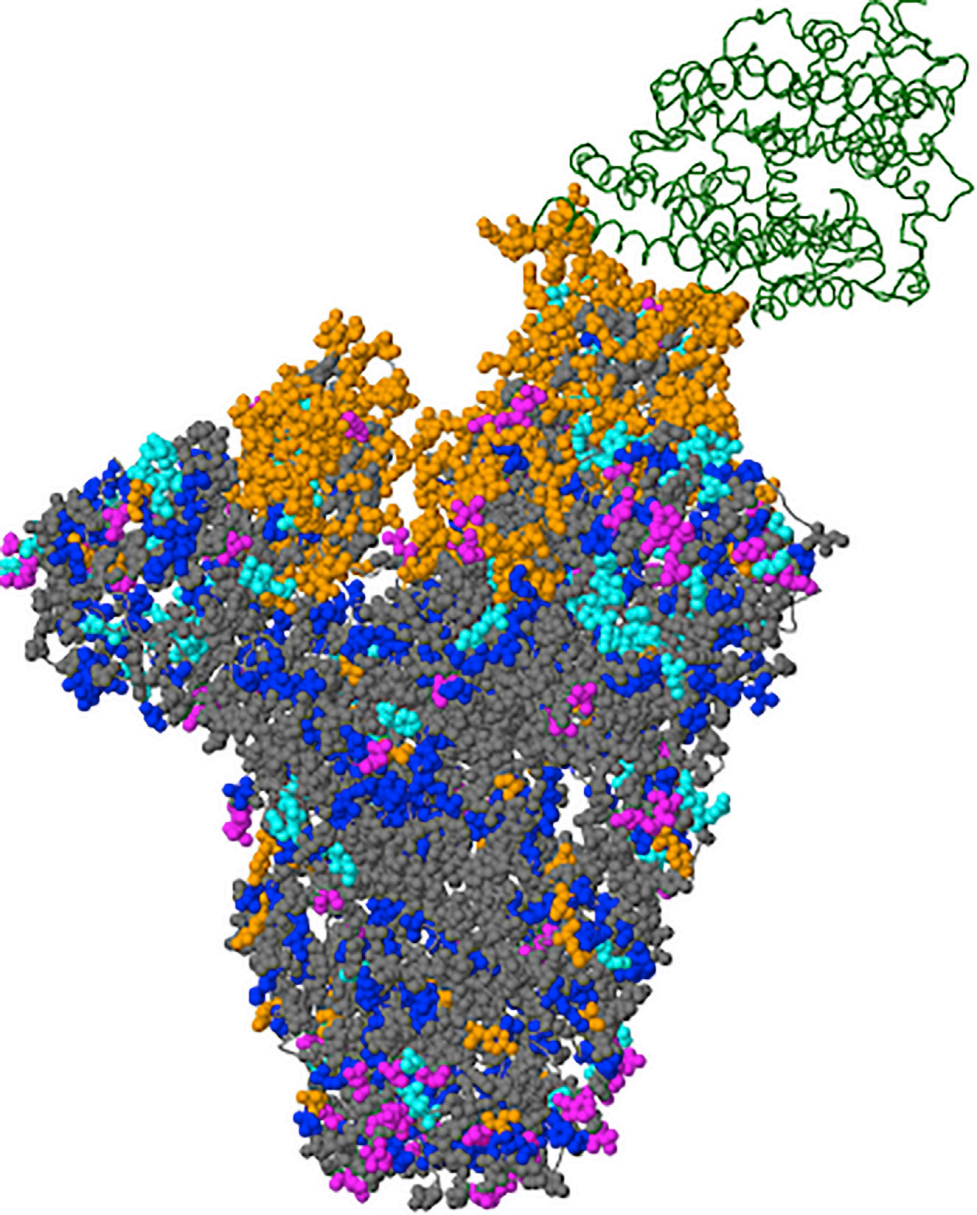
Spike glycoprotein complex Spike glycoprotein (PDB: 6ACJ, EM 4.2 Angstrom) in complex with ACE2 (green ribbon) showing the amino acid changes that occurred between Rousettus bat coronavirus (GenBank: AOG30822.1) and hCoV/Wuhan/WIV05/2019. The mutations in different strains are shown as colored balls.

An important property identified by SPIKES is JOND920102, which describes the degree of sequence differences among species, termed as ‘Relative mutability’ by Jones et al. (Jones et al., 1992). The relative mutability of amino acids demonstrates the amino acid changes that occur over time. Recurrent mutations are one of the crucial factors for the ongoing adaption of SARS-CoV-2 to the human host (van Dorp et al., 2020). To determine the significance of compositional changes in amino acids in S proteins from different strains of CoVs, we compared changes between Rousettus bat coronavirus (GenBank: AOG30822.1) and hCoV/wuhan/WIV05/2019 strain using the hCoV-19 spike glycoprotein mutation surveillance dashboard, GISAID. The results of the comparison are shown in Figure 5. Next, we used GISAID data statistics to examine the amino acid changes in the S protein that increased the infectivity in emerging new variants. The amino acid changes that increased the infectivity in different variants are shown in Table S1. Users may get more details on emerging variants from link.

**Figure 6:**
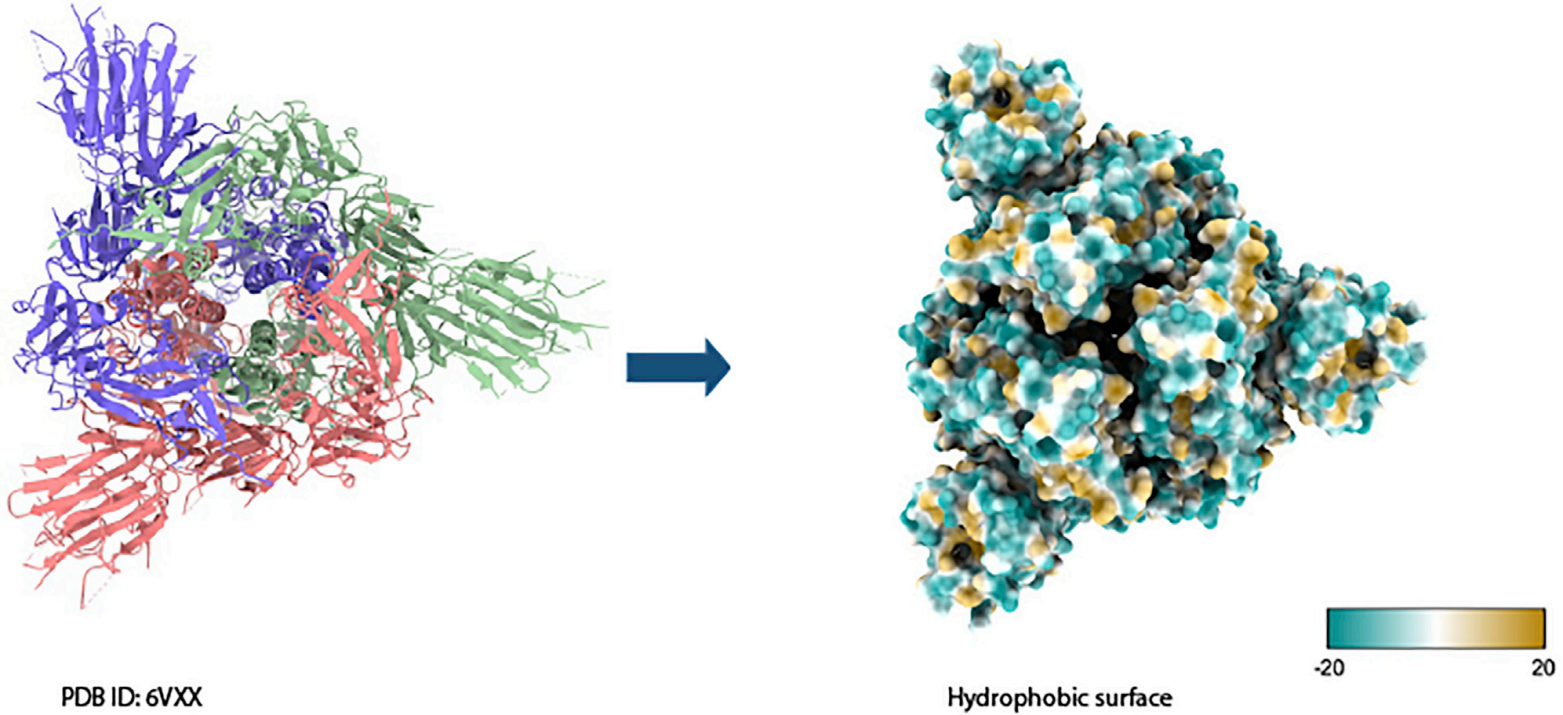
Secondary structure and surface hydrophobicity of spike protein 6VXX

From our previous analysis, we observed that the average hydrophobicity index for the hydrophobic amino acids of the S proteins in human host CoVs (0.18 ± 0.18) was slightly larger than that in animal CoVs (0.17 ± 0.14) (Yerukala Sathipati et al., 2022). To measure the surface hydrophobicity of an S protein, the Chimera protein visualization software can be used to view the protein structure. The surface hydrophobicity of an S protein is shown in Figure 6.

## Quantification and statistical analysis

The PCP comparison analysis was used to calculate the mean ± standard deviation of each PCP. The Student’s *t* test was used to compare group means between human and animal host CoVs; *p* < 0.05 indicates statistical significance. The LiBSVM package (Chang and Lin, 2011) was used to classify S proteins as being derived from human or animal host CoVs. Additional details can be found at link.

## Limitations

The SPIKES method described has some limitations. Using different feature selection algorithms and classifiers can produce contradictory predictions for the same dataset.

However, the robust SPIKES model was selected after 50 independent runs of IBCGA. We provided the stepwise procedure at link. Here, we only extracted the amino acid sequence-based features. However, the function of an S protein also could be influenced by the stoichiometry of its complex with the ACE2 receptor. Including a greater amount of S protein sequence data in the analysis could influence the selection of PCPs. It is a possibility that features selected using modified or alternative approaches might overlap, at least partially, with properties selected by SPIKES. Second, this study used data from GISAID until a certain period (May 2021); therefore, the inclusion of updated data may add to the significance of PCPs and amino acid compositions. Third, in vivo-based experimental validation and molecular docking studies could further strengthen the findings.

## Troubleshooting

### Problem 1

Protein sequences are not converting into AAindex properties.

### Potential solution

Ensure all sequences are in FASTA format. None of the amino acid sequences should contain uncertainties like ‘B, J, O, U, X, Z’ or other non-amino acid codons. FASTA files and aaindex.exe files should be in the same working directory.

### Problem 2

AA index conversion and scaling the FASTA sequences.

### Potential solution

Commands are case sensitive. Sequence files and executable files should be in the same working directory.

### Problem 3

Getting errors in modeling.

### Potential solution

Please follow the instructions on the Github page link for the input FASTA format and generating prediction results.

### Problem 4

How to extract the physicochemical property values from AAindex?

### Potential solution

Please use the AAindex matrix values provided in the link.

### Problem 5

How to perform mutational analysis?

### Potential solution

Please use the CoVsurver mutation analysis application link from GISAID to perform the analysis.

## Resource availability

### Lead contact

Further information and requests for resources and reagents should be directed to and will be fulfilled by the technical contact and lead contact, Ming-Ju Tsai (mingjutsai@hsl.harvard.edu) and Srinivasulu Yerukala Sathipati (sathipathi.srinivasulu@marshfieldclinic.org).

### Materials availability

This study did not generate new unique reagents.

## Data Availability

The protein sequence data used in this analysis is available at https://www.gisaid.org and https://www.ncbi.nlm.nih.gov. The pipeline of SPIKES and prediction model files are available at https://github.com/mingjutsai/SPIKES; https://doi.org/10.5281/zenodo.6502505. Additional supplemental information are available from Mendeley Data at https://data.mendeley.com/datasets/6s9wt7zzxz/1. https://doi.org/10.17632/6s9wt7zzxz.1.

## Supplemental information

Table S1. The amino acid changes that increased the infectivity in different variants, related to step-by-step method details step 15 and expected outcomes

## Footnotes

Supplemental information can be found online at https://doi.org/10.1016/j.xpro.2022.101460.
